# Editorial: Spondyloarthritis and omics

**DOI:** 10.3389/fimmu.2024.1475294

**Published:** 2024-08-14

**Authors:** Xiu-Feng Huang, Zhixiu Li

**Affiliations:** ^1^ Zhejiang Provincial Clinical Research Center for Pediatric Disease, The Second Affiliated Hospital and Yuying Children’s Hospital of Wenzhou Medical University, Wenzhou, Zhejiang, China; ^2^ School of Public Health and Emergency Management, Southern University of Science and Technology, Shenzhen, Guangdong, China

**Keywords:** spondyloarthritis, ankylosing spondylitis, OMICS, genetics, immunity

Spondyloarthritis (SpA) is a collective term for a group of inflammatory rheumatic diseases affecting the axial skeleton and potentially the peripheral joints. Given its substantial burden on patients and healthcare systems, understanding the intricate pathophysiology of SpA has become a critical research focus. The advent of omics technologies-encompassing genomics, proteomics, and metabolomics-has ushered in a new era of SpA research, enabling a more holistic and systems-level understanding of disease mechanisms.

We have selected articles in this Research Topic to illustrate recent advances in the omics of spondyloarthritis.

The integration of genomic data has been pivotal in identifying susceptibility loci for SpA, with HLA-B27 being the most established genetic marker. Genome-wide association studies (GWAS) have identified thousands of variants associated with complex traits, but their biological interpretation often remains unclear. The application of Mendelian randomization, as exemplified in a study investigating the causal relationship between immune cells and ankylosing spondylitis (AS), has provided novel insights into the potential for immune cells to serve as both risk factors and protective factors in SpA (Qin et al.). This approach underscores the importance of considering the bidirectional relationship between genetic determinants and immune cell dynamics in the pathogenesis of SpA.

Establishing causality helps in understanding the underlying biological mechanisms and pathways that link diseases. This knowledge is crucial for developing targeted therapies and preventive measures. Mendelian randomization study presented in our selection has uncovered a significant causal link between acute/subacute iridocyclitis and an elevated risk of AS, particularly within the European population (Li et al.). This finding underscores the necessity to consider the characteristics of AS when diagnosing iridocyclitis, offering new avenues for early detection and intervention.

Moving beyond genetics, the proteomic landscape of SpA reveals a complex interplay of inflammatory mediators, cytokines, and signaling molecules that contribute to disease pathogenesis. Studies leveraging proteomics have identified potential biomarkers that reflect disease activity and severity, offering a glimpse into the intricate immunological processes at play. Moreover, the exploration of the microbiome’s role in SpA, particularly through Mendelian randomization analyses, has suggested a causal link between gut microbiota and SpA (Tang et al.). This research opens avenues for investigating the gut-joint axis as a potential therapeutic target, aligning with the growing interest in the field of psychobiotics and their impact on rheumatic diseases.

The exploration of non-coding RNAs in AS, as discussed in a featured mini-review, opens up a new frontier in our understanding of the disease’s molecular mechanisms (Fang et al.). The regulatory roles of long non-coding RNAs (lncRNAs), circular RNAs (circRNAs), and micro RNAs (miRNAs) in AS are particularly highlighted, emphasizing their potential as diagnostic markers and therapeutic targets. This review brings to light the intricate networks of competing endogenous RNAs (ceRNAs) and their implications in inflammation, osteogenic differentiation, and cell death in AS.

The collective findings from these studies advocate for a personalized medicine approach to SpA management. The heterogeneity of SpA necessitates tailored interventions, with the omics data providing the granularity required to stratify patient populations and predict individual responses to treatment. As we stand on the cusp of precision medicine, the integration of multi-omics data holds the promise of uncovering the etiological underpinnings of SpA and facilitating the development of targeted therapies.

In conclusion, the synthesis of genetic, proteomic, and metabolomic data within the context of SpA research has propelled our understanding of this multifactorial disease. The studies featured in this Research Topic highlight the power of omics technologies in unraveling the complex interplay between genetics and immunity. As we continue to explore the depths of the omics landscape, we edge closer to deciphering the enigmatic nature of SpA, offering hope for improved diagnostics and therapeutics that can alleviate the burden of this debilitating disease.

